# An Item Response Theory Analysis of *DSM-5* Heroin Use Disorder in a Clinical Sample of Chinese Adolescents

**DOI:** 10.3389/fpsyg.2019.02209

**Published:** 2019-10-10

**Authors:** Hongmei Yang, Fu Chen, Xiaoxiao Liu, Tao Xin

**Affiliations:** ^1^Faculty of Psychology, Beijing Normal University, Beijing, China; ^2^Collaborative Innovation Center of Assessment toward Basic Education Quality, Beijing Normal University, Beijing, China

**Keywords:** heroin use disorder, substance use disorders, *DSM-5*, adolescents, item response theory, validity

## Abstract

The study examined the dimensionality and psychometric properties of *Diagnostic and Statistical Manual of Mental Disorders, 5th Edition* (*DSM-5*) criteria for heroin use disorder in a clinical sample of Chinese adolescents using item response theory approach. We examined 168 adolescents aged 14.8–17.9 years, who were in treatment for heroin use disorder. A two-parameter logistic item response theory model was conducted to examine the severity and discrimination of *DSM-5* criteria for heroin use disorder. Differential item functioning across age and ethnicity was assessed. Results supported the hypothesis that the *DSM-5* criteria for heroin use disorder were arrayed an underlying unitary dimension of severity in clinical adolescents. All the items exhibited good discriminatory power in distinguishing between clinical adolescent heroin users. Although three criteria exhibited measurement non-invariance at the item level, the overall *DSM-5* heroin use disorder diagnostic criteria set appears to achieve measurement invariance at the scale level.

## Introduction

Heroin is a highly addictive opioid drug with a high risk of overdose and death for users. A trend study from 2010 to 2014 revealed that approximately 20% of emergency department visits attributed to heroin overdose, and each year, these numbers were rising (Queeneth et al., 2019). Studies also revealed that nearly 8,986 adolescents have died from heroin overdose in the past 18 years in the US (Centers for Disease Control and Prevention, 2015). Studies using national data from large-scale substance use surveys indicated that adolescent heroin use is approximately 2% in the US (Brighthaupt et al., 2019). Considering that adolescent heroin use continues to be a public health globally, studying heroin use disorder in adolescents is critical. However, the lack of literature around the validity of adolescent heroin use disorder represents an important limitation in our understanding of adolescents with heroin use.

Since the publication of the *Diagnostic and Statistical Manual of Mental Disorders, 5th Edition* (*DSM-5*) (American Psychiatric Association, 2013), many studies have examined the psychometric properties of the *DSM*-*5* diagnostic criteria for substance use disorders in adult users using item response theory (IRT). These studies involved the use of alcohol (e.g., Mewton et al., 2011; Caetano et al., 2016), cannabis (Hasin et al., 2012), khat (Duresso et al., 2016), prescription opioids (Castaldelli-Maia et al., 2016), and cocaine (Hasin et al., 2012). These studies are of great importance because they testified to the validity of *DSM-5* diagnoses and disorders in the samples of users. Furthermore, the studies identified a unidimensional structure in the *DSM-5* criteria for substance use disorders using adult samples.

However, the previous studies often used samples of adults with alcohol use disorder in the general population to explore the dimensionality and psychometric properties of *DSM-5* criteria (e.g., Mewton et al., 2011; Caetano et al., 2016). Few studies have examined *DSM-5* criteria for heroin use disorder (HUD), especially in clinical settings. Within the *DSM* system of nomenclature, the criteria for illicit drug use are obtained largely based on the criteria for alcohol use disorder (Edwards and Gross, 1976). Compared to alcohol, illicit drugs, such as heroin, which have distinct pharmacological and cultural properties, are still under research (Hughes, 2006; Lynskey and Agrawal, 2007). Furthermore, clinical samples may represent the most severe end of the severity spectrum. Room (1980) argued that clinical samples qualitatively and quantitatively differ from samples from the general populations.

Globally, heroin use is an urgent concern, as the prevalence of heroin use disorder has increased rapidly (Martins et al., 2017). Unfortunately, we only found one clinical study (Hasin et al., 2012) that directly addressed *DSM-5* HUD criteria. The study found *DSM-5* criteria for HUD to have a unidimensional structure in a sample of 364 adult heroin patients. In addition, differential item functioning (DIF) by sex, age, and race/ethnicity was not found, whereas DIF by mood disorders was detected for heroin criteria.

Additionally, it is unclear whether the findings obtained from adults can be generalized to adolescents. Some researchers have argued that the operational concepts of the *DSM-IV* diagnostic criteria for substance use disorders appear to function well in adolescent users (Crowley, 2006). However, other studies suggested that, compared to adults, adolescents may exhibit different endorsement patterns (Martin et al., 2006; Hartman et al., 2008). Therefore, it is worth determining whether the *DSM*-*5* substance use disorders criteria can be suitably used in adolescent samples.

To date, *DSM-IV* or *DSM-5* criteria for HUD in adolescents have yet to be subjected to IRT analysis. Nevertheless, some studies have already examined the dimensionality and psychometric properties of the *DSM-IV* diagnostic criteria in adolescent users for substance other than heroin, such as alcohol and cannabis (Martin et al., 2006; Harford et al., 2009), prescription opioids (Wu et al., 2009b), hallucinogens (Wu et al., 2010), and inhalants (Perron et al., 2010). For instance, using IRT analysis, Martin et al. (2006) characterized the dimensionality and psychometric properties of *DSM-IV* diagnostic criteria for alcohol use disorder and cannabis use disorder among 472 adolescents from an addiction treatment program and found evidence of DIF in the assessment of alcohol use disorder and cannabis use disorder. Moreover, in an analysis of prescription opioid use disorders among adolescent users, Wu et al. (2009b) found evidence for the unidimensionality of the data using the IRT model; however, some items exhibited DIF by sex and race/ethnicity. Other studies have provided evidence for DIF across by racial/ethnic in adolescent substance users (Wu et al., 2009b). It is well known that Chinese ethnic minority groups are very diverse, but the total population of each ethnic group is far smaller than that of the Han Chinese. Therefore, it was important to consider the DIF across ethnicity, but only Han vs. minorities in Chinese sample.

IRT analysis of substance use disorders or other psychological disorders is a predominant method for measuring overall and individual criterion severity for diagnosis (Lane et al., 2016). IRT examines the endorsement patterns of each individual, allowing that different criteria may not have equal “weight” in predicting severity, rather than simply considering the criterion counts. Some particular criteria may be endorsed by severe users, whereas other criteria may be endorsed by mild users. Furthermore, using IRT analysis, studies have characterized dimensionality and psychometric properties to explore the utility and validity of diagnostic criteria. To study the utility and validity of diagnostic criteria, it is essential to characterize their psychometric properties (Nelson-Gray, 1991).

In brief, few studies have investigated the criteria for substance use disorders in *DSM-IV* or *DSM-5* in clinical samples using IRT models. Studies on adolescent clinical samples are even less common, particularly for heroin. However, the current epidemic of HUD in adolescents is a growing problem with devastating consequences for adolescents and their families (Sharma et al., 2016). To date, little is known about the dimensionality and psychometric properties of *DSM-5* diagnostic criteria for HUD in adolescents, especially in clinical samples. Compared to European countries or the United States, assessments of *DSM-IV* and *DSM-5* criteria for substance use disorders in Asian countries are limited. Additionally, because borders the Golden Triangle known for drug production and trafficking, China has one of the highest rates of heroin consumption, even though heroin use is prohibited and criminalized.

Therefore, the present study used IRT analysis to examine the dimensionality and psychometric properties of lifetime *DSM-5* HUD criteria in a clinical sample of Chinese adolescents and determine the endorsement patterns related to age and ethnicity. The study addressed the following questions: (1) Can IRT be used to analyze *DSM-5* HUD criteria? Specifically, we will examine the assumptions of IRT about unidimensionality and local independence. (2) Does the *DSM-5* HUD criteria exhibit good discriminatory power in distinguishing between clinical adolescent heroin users? (3) Does the DIF exhibit on some co-variables, such as age and ethnicity?

## Materials and Methods

### Sample

The data were collected from October to November in 2016. Participants were recruited from two drug rehabilitation centers in western China; one center was for males, and the other center was for females. The participants were recruited by squadron leaders of each treatment group in drug rehabilitation centers. All the squadron leaders got the recruitment information from the political commissar of the drug rehabilitation center. Interested squadron members of treatment group signed up for participation. To be eligible, participants must have used heroin and completed detoxification. All cases in the data were de-identified. Given that all the adolescents were younger than 18 years old, we spoke with their parents either in person or by telephone to ask for permission before recruiting them. The research protocol, including informed consent procedures, was approved by the Ethics Review Committee of the Beijing Normal University. The ages of the participants ranged from 14.8 to 17.9 years with a median age of 16.96 years. About 8.9% of the participants were female, and 67.9% were Han Chinese.

### Measures

Participants were assessed for HUD with a paper-assisted self-interviewing methodology to increase the validity of participants’ reports of heroin use. Questions based on *DSM-5* criteria were shown on a piece of paper and read out loud by the administrator. The participants were required to respond directly on the paper. Using a criteria count as a severity indicator of diagnoses, HUD was classified into three levels: mild (two to three criteria), moderate (four to five), and severe (six or more). Demographic variables in this study included participants’ age, sex, and ethnicity.

### Statistical Analysis

IRT assumes that the items and the latent trait can be connected through an appropriate IRT model, such as the two-parameter logistic (2PL) model for binary items in this study. The item response function for the 2PL model is as follows:

$$P\left(x_{ij}=1|\vartheta \right)=\frac{\exp\left(a_{j}\left(\vartheta_{i}-b_{j}\right)\right)}{1+\exp\left(a_{j}\left(\vartheta_{i}-b_{j}\right)\right)}\text{,}$$

where *ϑ* is the latent trait severity; *x_ij_* is the observed score of item *j* for person *i*; *b* is severity parameter (also named difficulty parameter) which is proportion of participants who answer item correctly or endorse the item, and *a* is discrimination parameter which is intended to discriminate participants with differing latent trait severity around the item’s threshold. According to Baker (2001), values of the difficulty parameter seen in practice is −2.80 to +2.80, but the theoretical range is −∞ to + ∞. Baker (2001) also suggested the range of values of the discrimination parameter is 0 to + ∞, and values larger than 1.7 can be considered as very high.

The 2PL IRT model is similar to binary factor analysis (CFA) model. Studies have suggested a general conversion formula to convert CFA model parameters to 2PL IRT parameters (Kamata and Bauer, 2008). The reader is referred to articles, such as Kamata and Bauer (2008) and Takane and De Leeuw (1987) for descriptions of the binary CFA model parameters and 2PL IRT model.

In the current study, CFA was conducted using the weighted least squares means and variance adjusted (WLSMV) estimation procedure by Mplus 6.11. To evaluate the local independence assumption of IRT model, we conducted local dependence analysis using *G*^2^ statistics. Then, we examined the relationship between participants’ responses to an item (criterion) and the severity of their heroin involvement using a 2PL IRT model (Reise and Waller, 2003). In order to examine whether the *DSM-5* heroin criteria functioned similarly across groups, we performed a DIF analysis for the criteria. The DIF analysis examined whether the severity parameters and the discrimination parameters for each criterion differed significantly in difference subgroups. The current study used the item response theory likelihood ratio test (IRT-LR) for detecting DIF within an IRT framework (Thissen et al., 1986). The item exhibits DIF when *b* parameter or *a* parameter of a criterion differs significantly across subgroups, after controlling for the latent trait being measured. The DIF covariates included age (≤ or > the median, 16.96 years) and ethnicity (minorities vs. Han). The IRT analysis was conducted by the ltm package (Rizopoulos, 2006) and mirt package (Chalmers, 2012) in R.

## Results

### Prevalence and Unidimensionality

**Figure 1:**
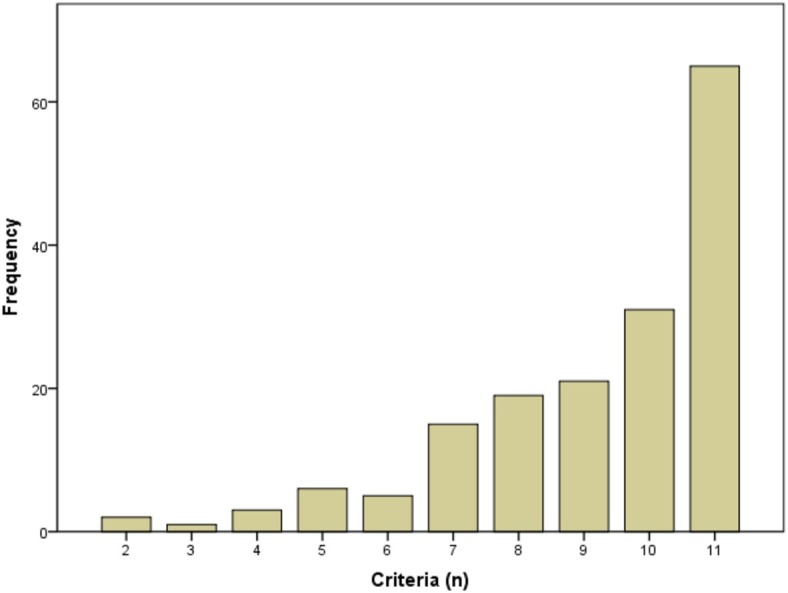
Distribution of total counts of criteria.

Based on the criteria in *DSM-5*, all participants were diagnosed with HUD. Specifically, 92.8, 5.4, and 1.8% were diagnosed with severe, moderate, and mild HUD, respectively. The distribution for the total counts of the criteria is shown in Figure 1. The lifetime prevalence of each criterion ranged from 73.2% for the *Craving* criterion to 91.1% for the *Social/interpersonal* criterion (see Table 1).

**Table 1:** The prevalence and IRT parameters of the *DSM-5* HUD criteria (*N* = 168).

Items	DSM-5 definition	Prevalence (%)	Severity	Discrimination
Larger/longer	Often using more or for a longer period than intended	79.2	−1.19	1.63
Quit/control	Persistent desire or unsuccessful attempts to quit or cut down heroin use	85.1	−2.07	0.99
Time spent	Lots of time spent using, obtaining, or being affected by heroin	85.7	−1.61	1.54
Craving	Cravings (the presence of a strong desire to use)	73.2	−0.95	1.50
Neglect roles	Frequent intoxication leading to failure to fulfill major role obligations	87.5	−1.48	2.16
Social/interpersonal	Continued use despite social or interpersonal problems caused or exacerbated by use	91.1	−1.68	2.34
Activities given up	Important social activities given up or reduced due to heroin use	86.3	−1.89	1.22
Hazardous use	Recurrent use when it is physically hazardous	82.1	−1.56	1.25
Physical/psychological	Continued use despite physical/psychological problems caused or exacerbated by use	86.9	−1.58	1.76
Tolerance	Need to consume more to achieve same effect; decreased effect with same amount	86.3	−1.75	1.38
Withdrawal	Signs of withdrawal syndrome; use to avoid withdrawal	79.2	−1.53	1.06

As for the dimensionality, the one-factor model was found to fit the HUD criteria in *DSM-5* well, as *χ*^2^ = 45.78, df = 44, *χ*^2^/df = 1.04; *p* = 0.40, CFI = 0.993; TLI = 0.991; SRMR = 0.110, RMSEA = 0.016 (90% CI: 0.000, 0.055), *p* for test of close fit (RMSEA <0.05) is 0.916. The results of the CFA analysis supported the hypothesis the HUD symptoms were arrayed along a unidimensional construct.

Local independence is a critical assumption in IRT since bias would be produced if the assumption deviated. Results showed that *p*’s for test of *G*^2^ statistics ranged from 0.09 to 0.98. That is to say, all of the *p*’s were larger than 0.05 (Table 2). Therefore, local independence assumption was confirmed.

**Table 2:** Local dependence indexes (*G*^2^) for item pairs of the *DSM-5* HUD criteria (*N* = 168).

Items	1	2	3	4	5	6	7	8	9	10	11
1. Larger/longer		0.24	0.33	0.84	0.59	0.70	0.44	0.62	0.72	0.17	0.09
2. Quit/control	−1.41		0.63	0.89	0.56	0.71	0.93	0.06	0.70	0.77	0.09
3. Time spent	0.94	−0.23		0.85	0.48	0.12	0.63	0.69	0.30	0.19	0.21
4. Craving	0.04	−0.02	0.03		0.47	0.81	0.30	0.60	0.29	0.42	0.80
5. Neglect roles	−0.29	0.34	−0.49	0.53		0.49	0.92	0.95	0.85	0.15	0.71
6. Social/interpersonal	−0.15	0.13	−2.44	0.06	−0.49		0.31	0.94	0.46	0.52	0.53
7. Activities given up	−0.61	−0.01	0.23	−1.08	−0.01	−1.03		0.56	0.98	0.82	0.51
8. Hazardous use	−0.24	−3.71	−0.16	−0.28	0.01	0.01	0.34		0.50	0.27	0.80
9. Physical/psychological	−0.13	−0.15	−1.10	−1.15	−0.04	0.56	0.01	0.45		0.46	0.13
10. Tolerance	1.86	0.09	−1.71	−0.65	−2.05	−0.42	0.05	−1.22	−0.54		0.53
11. Withdrawal	−2.81	2.85	1.56	0.06	0.14	−0.40	−0.43	−0.06	−2.26	0.39	

*Lower triangle: G^2^; upper triangle: p’s; degrees of freedom was 1 in each item pair*.

### Item Response Theory Results

The estimates of the severity and discrimination parameters are shown in Table 1. For the sample used in this study, the estimates of the severity parameter were on the lower end of the severity continuum for heroin involvement. The item severity parameter measures the degree of severity of heroin use problems, ranging from −2.07 to −0.95. More specifically, the *Craving* and *Larger/longer* criteria showed the greatest severity levels, whereas the *Quit/control* and *Activities given up* criteria showed the lowest severity levels in this study. Globally, the estimates of the severity parameters were on the lower end of the severity continuum for heroin involvement in this study. The discrimination parameter estimates the precision with which an item distinguishes between participants with levels of the latent trait above and below the item’s severity. The item discrimination estimates varied from 0.99 to 2.34, indicating that the criteria in *DSM-5* have good discriminatory power in distinguishing between adolescent heroin users with different severities. Specially, the *Social/interpersonal* and *Neglect roles* criteria showed the highest discrimination ability, and the *Quit/control* and *Withdrawal* criteria showed the lowest discrimination (see Table 1).

**Figure 2:**
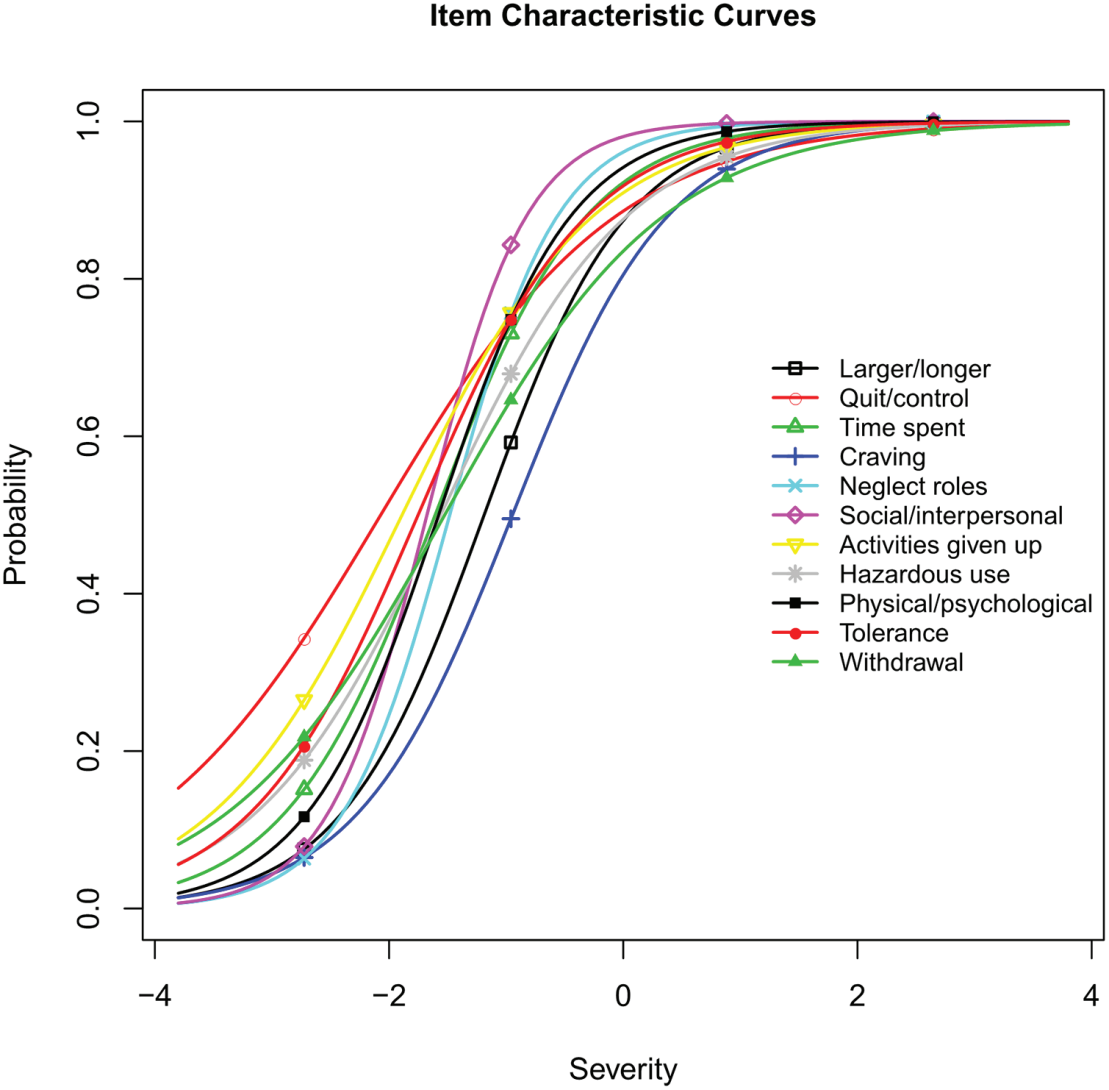
Item characteristic curves for *DSM-5* heroin use disorder criteria.

The item characteristic curves (ICCs) using these estimates are depicted in Figure 2. ICCs are graphical displays with item severity and discrimination parameters. It should be noted that the *Craving* criterion (severity = −0.95, discrimination = 1.50), which had the greatest severity (indicating lower prevalence), demonstrated a moderate discrimination level compared to the other criteria.

**Figure 3:**
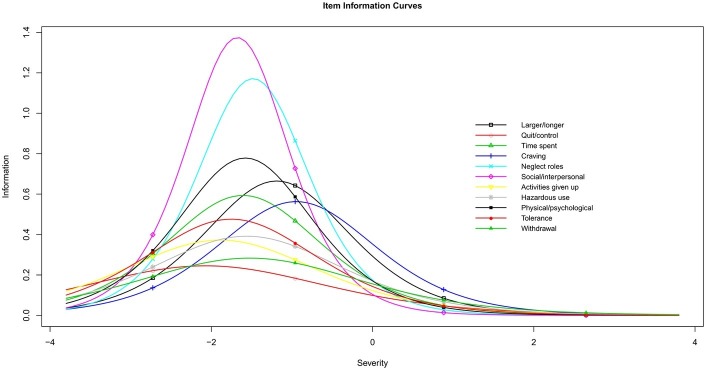
Item information curves for *DSM-5* heroin use disorder criteria.

To show where the criteria maximized the measurement accuracy for the participants, the item information curves were depicted (Figure 3). According to the item information curves, most of the HUD criteria maximized the measurement accuracy for the participants with severity estimates of approximately −1.5. The severity estimates at which each criterion maximized the measurement accuracy ranged from −2.07 to −0.95. More specifically, the *Social/interpersonal* criterion provided the most information, and the *Quit/control* criterion provided the least information. Specifically, the largest information provided by the *Social/interpersonal* criterion was 1.37, and the corresponding HUD severity was −1.68. However, the largest information for the *Quit/control* criterion was 0.23, and the corresponding HUD severity was −2.07.

**Figure 4:**
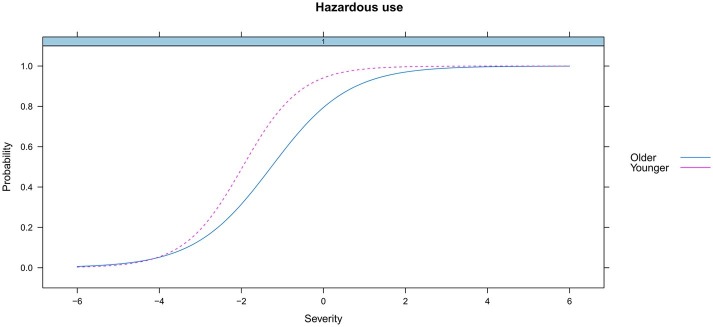
DIF for hazardous use by age.

**Figure 5:**
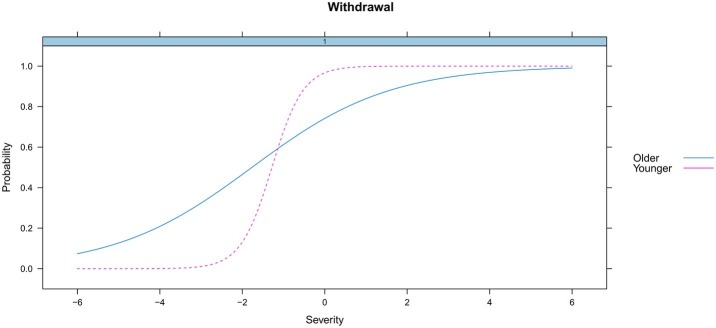
DIF for withdrawal by age.

**Figure 6:**
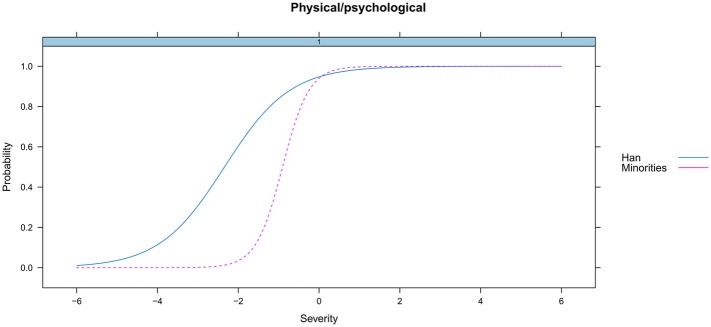
DIF for physical/psychological by ethnicity.

We examined the DIF for age (≤ or > the median, 16.96 years) and ethnicity (minorities vs. Han Chinese) across groups. Results showed that there existed no differential functioning for the total score (the underlying HUD trait) by age (*p* = 0.30) or ethnicity (*p* = 0.31). However, differential functioning was detected for 3 of the 11 criteria for heroin use. Specifically, the DIF analysis found that two criteria (*Hazardous use* and *Withdrawal*) functioned differently across age (Figures 4, 5), and there were significant differences for one criterion (*Physical/psychological*) across ethnicity (Figure 6). Younger participants were more likely than older participants endorse the *Hazardous use* criterion (Figure 4). Furthermore, younger participants were more likely to endorse the *Withdrawal* criterion at high levels of HUD severity, while those older participants were more likely to endorse the *Withdrawal* criterion at low levels of HUD severity (Figure 5). In addition, the *Physical/psychological* criterion was also more likely to be endorsed by the Han users than by minority users (Figure 6). These results suggested that these three criteria differed significantly in different samples.

## Discussion

To our knowledge, this is the first study that specifically focused on the *DSM-5* criteria for HUD in a clinical sample of adolescent heroin users. We explored the dimensionality and psychometric properties of the *DSM-5* criteria for HUD within the IRT framework. Specifically, we examine the unidimensionality, severity, and discrimination levels and differential functioning for the *DSM-5* HUD criteria.

As expected, the HUD criteria measured a single underlying construct in this clinical sample of adolescent heroin users. This finding echoes the previous studies focusing on other substance use disorders in adults or adolescents (Wu et al., 2009b; Hasin et al., 2012; Saha et al., 2012; Caetano et al., 2016; Castaldelli-Maia et al., 2016; Duresso et al., 2016).

As stated by some authors (Reise and Waller, 2009; Thomas, 2011), because clinical samples consist of individuals with more severe conditions than those in the general population, the severity of individual HUD criteria should be lower than that found in the general population. In this study, all of the severity parameters were negative, which is similar to the findings of previous studies using the *DSM-5* HUD criteria (Hasin et al., 2012) or using the *DSM-IV* cocaine and opioid use disorder criteria (Wu et al., 2009a) in clinical adults. Our study also found that *Quit/control* was the least severe criterion, which is consistent with the findings of a previous study using a sample of adults (Hasin et al., 2012). This suggests that many heroin users often experience “persistent desire or unsuccessful efforts to cut down or control use.” Regarding item discrimination, generally, all criteria exhibited good discriminatory power in distinguishing between clinical adolescent heroin users. The range of the discrimination parameters was also consistent with that in a study on opioid use disorder in clinical adults (1.19–2.18) (Wu et al., 2009a).

Overall, the HUD criteria appeared to perform very similarly across age and ethnicity. There was no evidence of differential functioning for the total score (the underlying HUD trait) by age or ethnicity, which is similar to the findings of studies on other substance use disorders in adults and adolescents (Mewton et al., 2010; Hasin, 2015).

Similar results were reported that the *Hazardous use* criterion decreased with increasing age in adult populations. These studies involved the use of alcohol (Kahler and Strong, 2006; Harford et al., 2009) and cannabis (Martin et al., 2006; Mewton et al., 2010). Moreover, DIF by age was found in the *Withdrawal* criterion in our study, which is consistent with finding that DIF existed for the *Withdrawal* criterion by different age subgroups in adult alcohol users (Saha et al., 2006). In addition, Wu et al. (2009b) found that the *Physical/psychological* criterion had race/ethnicity DIF in adolescent opioid users, which is consistent with the finding of DIF by ethnicity for the *Physical/psychological* criterion in our study. In brief, it should be noted that the significant DIF detected by age and ethnicity in these three criterion suggests that they measures something other than the HUD construct. Generally, the identification of DIF suggests that the criterion is evaluating something other than the underlying construct it is intending to measure (Mewton et al., 2010).

A few study limitations should be noted. First, as with all other substance use disorders studies, the information obtained from participants is vulnerable because of self-report bias. However, since the participants reported a high prevalence of each HUD criterion in our study, we believe that self-report bias did not significantly affect the results. Second, because our study used a small sample of adolescents, the results should be interpreted with caution. Finally, since the sample included few females, the DIF by sex could not adequately be examined in our study. The percentage of females was 8.9% in our study, while data from the China National Narcotic Control Committee showed that females accounted for 14.4% of the total drug users in 2016.

In conclusion, we explored the psychometric properties of *DSM-5* diagnostic criteria for HUD in a clinical sample of adolescents. The results showed that the overall HUD diagnostic criteria set in *DSM-5* is of high psychometric quality. The construct of heroin use disorder using *DSM-5* criteria appears valid, and its performance is consistent with those of other substance use disorders. However, differential item functioning was found by ethnicity and age for three criteria, suggesting that these three criteria should be used with caution for certain samples.

## Data Availability Statement

The datasets generated for this study will not be made publicly available because the policy of drug rehabilitation centers do not permit. Requests to access the datasets should be directed to the corresponding author.

## Ethics Statement

The studies involving human participants were reviewed and approved by the Ethics Review Committee of Beijing Normal University. Written informed consent to participate in this study was provided by the participants’ legal guardian/next of kin.

## Author Contributions

All authors have materially participated in the research and the manuscript preparation. HY and TX designed the study and wrote the protocol. HY conducted statistical analysis of the data and wrote the first draft of the manuscript. FC and XL contributed to the final manuscript. All authors have approved the final manuscript.

### Conflict of Interest

The authors declare that the research was conducted in the absence of any commercial or financial relationships that could be construed as a potential conflict of interest.
